# Abnormal Muscle Activity and Variability Before, During, and After the Occurrence of Freezing in Parkinson's Disease

**DOI:** 10.3389/fneur.2019.00951

**Published:** 2019-09-03

**Authors:** Hiram Cantú, Julie Nantel, Michelle Millán, Caroline Paquette, Julie N. Côté

**Affiliations:** ^1^Departamento de Ingeniería Biomédica, Vicerrectoría de Ciencias de la Salud, Universidad de Monterrey, San Pedro Garza García, Mexico; ^2^Occupational Biomechanics and Ergonomics Laboratory, Michael Feil and Ted Oberfeld/CRIR Research Centre, Jewish Rehabilitation Hospital, Laval, QC, Canada; ^3^Department of Kinesiology and Physical Education, McGill University, Montreal, QC, Canada; ^4^School of Human Kinetics, University of Ottawa, Ottawa, ON, Canada; ^5^Centre for Interdisciplinary Research in Rehabilitation, Montreal, QC, Canada

**Keywords:** Parkinson's disease, freezing, freezing of gait, freezing episodes, electromyography, amplitude, variability, mutual information

## Abstract

Freezing of gait (FOG) is often experienced in advanced stages of Parkinson's disease (PD) and can lead to an increased risk of falls. Although spatiotemporal characteristics of FOG are well-described, their underlying neuromuscular mechanisms remain poorly understood. Several studies have demonstrated an abnormal activation of distal muscles of the lower limb and coordination impairments during gait in people with PD (pwPD). However, few have investigated how various characteristics of electromyograms (EMGs) change before, during and after a freezing episode (FE). Our objective was to quantify changes in proximal and distal leg muscle activity associated with FEs. In this study, 12 pwPD, confirmed as freezers, performed a repetitive stepping-in-place task used to elicit FE. Surface EMGs were collected from proximal [rectus femoris and biceps femoris (BF)] and distal [tibialis anterior (TA) and gastrocnemius medialis (GM)] muscles. Data epochs of 500 ms were extracted from EMG time series at four different periods: baseline, 2 s before a FE, during a FE, and 2 s after a FE. For each epoch, EMG amplitude [root-mean-square (RMS)], variability [coefficient of variation (CoV)], and inter-muscle functional connectivity (mutual information) were quantified. Results from the analysis of 21 FEs show a significant main effect of Period for EMG amplitude in bilateral TA and in the least affected GM (*p* < 0.01), with decreased activation before freezing that remained low during and after the FE. On the other hand, a main effect of Period was also found in bilateral BF muscles (*p* < 0.01) but with increased activation before freezing that was generally sustained during and after FE. Main effects of Period were also found for all measures of variability, except for the least affected GM, showing reduced variability during the FE that returned to baseline in all muscles except both TA. Moreover, an increase in functional connectivity between the least affected distal muscles was seen before the FE. Our findings confirm that many characteristics of EMG patterns of both distal and proximal leg muscles change throughout periods of a FE, suggesting both impairment and adaptive strategies from proximal muscles.

## Introduction

Parkinson's disease (PD) is a degenerative, progressive motor system disorder caused by the loss of nigro-striatal neurons. PD is associated to various impairments in motor function. In addition to the main PD symptoms of bradykinesia, tremor, rigidity, and postural instability, individuals with PD often experience gait disturbances, such as freezing of gait (FOG) (1–3). Mostly present in the latest stages of the disease, FOG has been described as the inability to complete a step regardless of the intention to move (4). Therefore, it is one of the most disabling symptoms of PD (5–8). Although some characteristics of FOG such as high-frequency, decreased step amplitudes, trembling-like leg movements, and gait festination, have been well-described (4, 7, 9), the causes of FOG remain unknown.

Surface electromyography (EMG) is a non-invasive technique primarily used, in clinical settings, to better understand the physiopathology of neuromuscular dysfunction as well as to diagnose and evaluate the progression of the motor dysfunction (10, 11). Many previous studies used EMG to quantify muscle activation and better understand the origin of disturbances associated with PD during the performance of various discrete upper and lower limb tasks. For instance, Sande de Souza et al. (12) found that people with PD (pwPD) have difficulties modulating the activity of agonist and antagonist muscles during elbow and shoulder flexion and extension motions. Moreover, they found that the slowness of movement seen in PD while performing this task was associated with low amplitude of muscle activity. Similarly, impaired motor excitability modulation was reported for the soleus and tibialis anterior (TA) in pwPD during voluntary dorsiflexion contractions (13).

Of all the motor functions studied using EMG in pwPD, cyclical activities such as gait are the most common. Many studies in pwPD analyzed EMG patterns during gait trials free of FE. For instance, Bailey and colleagues recently demonstrated asymmetry in the activation of the gastrocnemius lateralis in pwPD while walking (14). They found that this asymmetry was directly related to a lower score in the Unified Parkinson's Disease Rating Scale (UPDRS-III) and suggested that it might play an important role in postural stability impairments in pwPD (14). In line with these results, it was found that pwPD who experience FOG showed a reduced bilateral gait coordination (15), and that the gastrocnemius lateralis is active in gait phases where falls occur (16). Similarly, Albani et al. (17) found an increased TA activity in individuals with FOG when walking on a treadmill. However, the authors also found a reduction in gastrocnemius medialis (GM) activity, suggesting a complex interplay between ankle plantar- and dorsiflexors during gait affected by PD. In line with this result, another study suggested that GM activity might be a strong indicator and predictor of FOG, since a significantly reduced activity in the GM was found in pwPD screened as “freezers” compared to “non-freezers” (18). Taken together, while it is clear from these studies that freezers have an abnormal activation of TA and GM when walking, most of these studies focused on the EMG activity of the distal muscles of the legs (TA and GM), and little is known about how PD affect patterns of the knee and hip muscles during gait tasks.

In an effort to better understand the control mechanisms underlying motor disturbances in PD, other more complex measures of the electromyogram have been recently investigated. It was shown that during a ramped-up knee extension task, pwPD exhibited less changes in amplitude activation and muscle activity variability than a healthy control group (19). Moreover, the same research group showed that these more fixed activation patterns are even less variable during off-medication compared to on-medication periods (20). Although these studies suggest a relation between low motor variability and impairment, a recent study investigating the effects of physical therapy on lower limb muscle activity during gait in pwPD found that higher variability of gastrocnemius lateralis was related to higher dysfunction; however, their variability was calculated over the EMG signals' modulation indices, not the amplitude indices (14). Finally, indices of inter-muscle coordination have previously been used to study more complex coordination behavior and how it may be affected by PD. Egerton et al. showed an absence of coordination between the TA and GM muscles during a gait initiation task, with the TA showing little to no activation and the GM showing unusually prolonged activity (21). Nevertheless, aside from these studies, few have investigated inter-muscle coordination patterns during gait in pwPD before, during or after FEs.

Gait initiation impairments have been previously described in pwPD, especially in those who experience freezing. Halliday et al. (22) demonstrated a similar suspension of activity on the GM by the end of the postural phase, just before the transition from standing to walking, in groups of young and elderly people compared to a group of pwPD. However, in the majority of the cases of participants with PD there was no bilateral activation of TA during gait initiation. In line with these results, along with a decrease in EMG activity of muscles that contribute to gait, such as TA, GM, and vastus lateralis, authors described that pwPD who experience freezing only had unilateral activation of TA during gait initiation (23). Moreover, Schlenstedt et al. (24) recently observed a higher hip co-contraction between left and right tensor fasciae latae in a group of pwPD with freezing compared to a group of pwPD who did not experience freezing during anticipatory postural adjustments prior gait initiation. They also reported higher GM activity in pwPD with freezing compared to a healthy control group. Taken together, all these findings indicate that gait initiation deficits seen in PD are a consequence of impaired anticipatory postural adjustments that are characterized by abnormal timing and amplitude activation of postural muscles, including TA and GM.

Fewer, but some studies investigated ankle muscle control during gait trials in which freezing episodes (FEs) occurred. Nieuwboer and colleagues (25) showed reduced EMG activity in the strides prior to a FE. However, they reported unchanged EMG activation amplitude of the GM and increased amplitude of the TA, when EMG was normalized to burst duration. They also observed abnormal timing between TA and GM, characterized mainly both by their premature activation, but more prolonged bursts in the GM. Mezzarobba et al. (26) observed changes in the activation timing of the TA, GM, gluteus maximus, and gluteus medius three steps prior to FEs during gait. They concluded that FE results from a progressive disorganization in gait planning and execution rather than being a sudden event. Recently, a study introduced a method partly based on surface EMG measurements recorded from distal leg muscles to identify FEs (27). However, they only considered amplitude and timing characteristics of individual EMG signals, and they did not statistically assess how EMG characteristics changed before, during, and after freezing.

The general objective of this project was to better understand the muscular mechanisms underlying the occurrence of FEs in PD. In this paper, we sought to quantify muscle amplitude activation, variability, and inter-muscle coordination of lower limb muscles surrounding a FE. To our knowledge, this is the first study to investigate EMG characteristics other than amplitude, in rectus femoris, biceps femoris, TA, and GM muscles, in different periods of the freezing phenomenon, in pwPD experiencing FEs. We hypothesized that changes in amplitude, variability and inter-muscle coordination would occur at different phases relative to FEs.

## Materials and Methods

### Participants

A group of 26 pwPD was recruited from the Cummings Center for Seniors in Montreal, Quebec, and from the Quebec Parkinson Network to participate in this study. Participants were excluded from this study if they had: (1) any orthopedic, muscular, or neurological disorder other than PD, (2) mild cognitive impairment (score <26 on the Montreal Cognitive Assessment), (3) deep brain stimulation surgery, (4) were taking medication affecting balance other than the anti-parkinsonian medication, or (5) a history of diabetes. All participants performed an experimental protocol twice on separate days with a mean of 8.3 ± 3.7 days between sessions. Participants were on dopaminergic medication during the first session and off-medication for the second visit, abstaining from levodopa overnight (washout period ≥12 h). Both sessions were conducted at the same time of the day and under the same conditions to avoid within-day motor fluctuations. Ethical approval for this study was received from the Research Ethics Board of the Center for Interdisciplinary Research in Rehabilitation (CRIR) of Greater Montreal. All participants provided written, informed consent prior to participation.

### Clinical Evaluation

During the first session (i.e., in their best on-medication state), participants were clinically assessed by the same examiners: (1) medical history questionnaire, (2) Montreal Cognitive Assessment, (3) Unified Parkinson Disease Rating Scale Part 3 (UPDRS-III), (4) Hoehn and Yahr (H&Y) scale, and (5) Freezing of Gait Questionnaire (FOGQ). In order to assess the change in disease severity in their off-medication state, the H&Y scale and the UPDRS-III evaluations were administered again in the second session. Participants were further categorized as freezers (*n* = 12) if they answered experiencing at least once a month (score of 1 or greater) to the FOGQ question #3: “*do you feel that your feet get glued to the floor while walking, making a turn or when trying to initiate walking?”* and/or if freezing was observed in any testing session. For this reason, all participants were also included in the study, to account for the possibility that even those who self-identified as non-freezers could experience freezing during their visits.

### Experimental Protocol

All participants performed a repetitive stepping-in-place (SIP) task (28, 29) during both sessions to elicit freezing. The SIP task consists of raising the legs (hip and knee flexion) alternately from a standing position. During two 20 s practice trials, participants were instructed to perform the task as fast as possible but comfortable enough for the participants to be able to finish the entire experimental protocol. Cadence for each participant was set as the fastest of both practice trials in order to increase the likelihood of freezing occurrences (30, 31). A 10-beat auditory feedback from a metronome ensured that participants followed the prescribed cadence. The auditory feedback was presented prior to each trial, after which they were asked to memorize and perform the task following this rhythm. The experimental protocol included two trials of the SIP task, one of 30 s and one of 120 s. At all times, participants wore a harness attached to the ceiling that did not restrict natural movement but would catch them in case of a fall. None of the participants fell during the task.

### Data Acquisition

**Figure 1:**
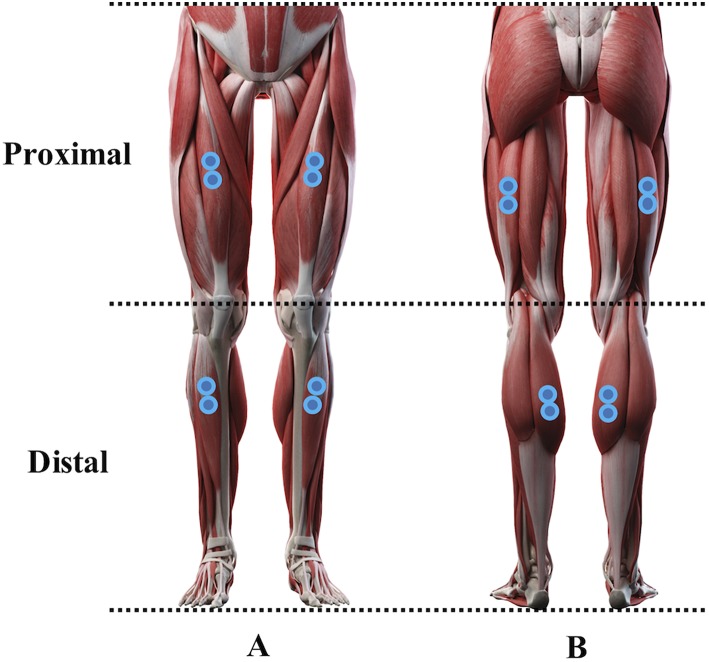
Anterior **(A)** and posterior **(B)** representation of the electrodes position over the muscles analyzed in the study. A pair of electrodes were placed over proximal muscles [bilateral rectus femoris **(A)** and biceps femoris **(B)**] as well as over distal muscles [bilateral tibialis anterior **(A)** and gastrocnemius medialis **(B)**] (Sebastian Kaulitzki/stock.adobe.com).

Surface electromyography (EMG) was acquired continuously during the 30 s and 120 s trials from 4 muscles bilaterally according to guidelines published previously (32) from the following muscles (see Figure 1):

- Proximal muscles:
  ° Rectus femoris (RF; 50% on the line from the anterior spina iliaca superior to the superior part of the patella).
  ° Biceps femoris (BF; 50% on the line between the ischial tuberosity and the lateral epicondyle of the tibia).

- Distal muscles:
  ° Tibialis anterior (TA; one third on the line between the tip of the fibula and the tip of the medial malleolus).
  ° Gastrocnemius medialis (GM; the most prominent bulge of the muscle).

A pair of electrodes was placed over each of the muscles belly, with center-to-center distance of 3 cm, parallel to the muscle fibers, and after careful preparation (skin cleaned with alcohol and shaved for better signal acquisition). A TeleMyo EMG measurement system (TeleMyo 900, Noraxon USA, Inc.) with an operating bandwidth of 10–500 Hz, an effective common mode rejection ratio of 130 dB DC, >100 dB at 60 Hz, a minimum of 85 dB throughout the operating bandwidth, and a fixed overall per-channel gain of 2,000 was used. EMGs were sampled at 1,000 Hz, digitally converted using a 16 bit A/D board over a ±10 V range, and stored for further analysis.

### Data Analysis

EMG signals of the eight muscles were filtered off-line using a dual-pass, fourth-order Butterworth filter, with a band-pass of 20–500 Hz, to remove any artificial noise created during the acquisition and analysis procedures. Signals were then full-wave rectified.

A freezing episode (FE) was defined as a minimum time of 0.5 s where participants were unable to completely lift a foot off the floor during the SIP task. If the time between two consecutive FEs lasted <2 s, it was considered as part of a long FE. For more information on the methodology followed to detect a FE, the reader is referred to Cantú et al. (33). In order to ensure that data analysis was done in a representative set of data, from all the FEs identified in the study, only those lasting >2 s and with at least 2 s of non-freezing occurrence before and after were chosen for further analysis.

**Figure 2:**
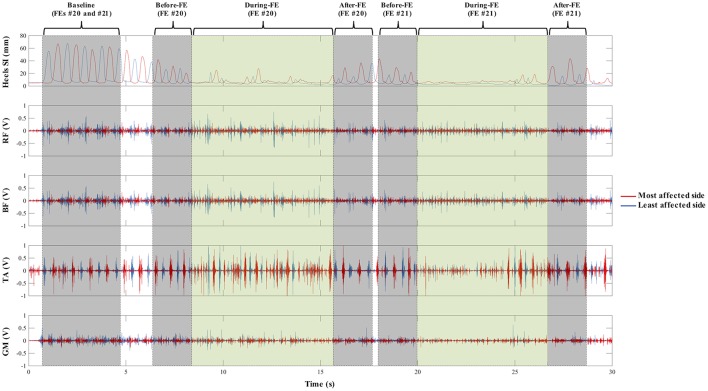
Graphic representation of the four periods extracted from each trial where a freezing episode (FE) was found: 4 s of normal performance of the stepping-in-place task at the beginning of the trial (Baseline), 2 s before the occurrence of the FE (Before-FE), during the FE (During-FE), and 2 s after the end of the FE (After-FE). A representative 30 s trial sample (from top to bottom) of bilateral lower limb movement (markers placed on the heels) in the superior-inferior (SI) direction, and of the activity of bilateral rectus femoris (RF), biceps femoris (BF), tibialis anterior (TA), and gastrocnemius medialis (GM) during the stepping-in-place (SIP) task. The red and blue lines represent the most and least affected sides, respectively. A total of two FEs (#20 and #21) were found in this trial. Kinematic data was obtained by using a motion capture system and was not analyzed for this paper. In both TA and least affected GM traces, a slight decrease activation in the times surrounding a FE is seen, especially During-FE compared to Baseline (more in FE #21). Additionally, a less variable EMG trace is seen for all muscles During-FE compared to the other periods, which is recovered by the end of the freezing episodes and After-FE.

Four periods were extracted from each trial of the SIP task in which a FE that was considered for analysis was identified:

- Baseline: 4 s of normal performance of the SIP task at the beginning of the trial.
- Before-FE: 2 s before the occurrence of the FE.
- During-FE: during the FE.
- After-FE: 2 s after the end of the FE (see Figure 2).

Each period was further partitioned in non-overlapping 500 ms windows. Because the During-FE varied in duration, the maximum number of 500 ms windows was fitted starting at the beginning of the FE. EMG variables (amplitude, variability, functional connectivity) were computed over each 500 ms window and were averaged over each period.

EMG amplitude was calculated by computing the root-mean-square (RMS) over the entire 500 ms window. Variability was obtained by computing the coefficient of variation (CoV, corresponding to the 500 ms signal's standard deviation divided by the mean). Inter-muscle coordination was assessed using a measure of functional connectivity among proximal (RF and BF) and distal (TA and GM) muscle pairs. The measure chosen to assess functional connectivity was mutual information (MI), a measurement similar to cross-correlation but that accounts for non-linear characteristics of each signal within a muscle pair and of their relationship. The MI outcome is a number that varies from 0 to 1, 0 indicating no connectivity and 1 complete connectivity within the muscle pair (34). It has been used more widely in the recent EMG literature studying conditions such as muscle fatigue, delayed onset muscle soreness (35), as well as to study coherence in brain patterns of different regions in various clinical conditions. All data analysis was performed using Matlab 2018b (MathWorks, Massachusetts, USA).

### Statistical Analysis

For statistical analysis, muscles were categorized according to the disease laterality (most affected side, least affected side) that was recorded from participants' clinical evaluations. Descriptive statistics of participants are presented as mean and SD. Tests of normality (Shapiro-Wilk test) were run for RMS, CoV, and MI of the eight muscles analyzed. Since the distribution of EMG amplitude (RMS) and variability (CoV) violated normality, these outcome measurements were compared across period by means of a non-parametric repeated measures Friedman test for equal sample sizes having as independent factor the within-participant condition of Period (Baseline, Before-FE, During-FE, and After-FE). Repeated measures ANOVA was conducted to compare the effect of Period on shared information and functional connectivity (MI) among muscle pairs of the leg. If sphericity was violated after running Mauchly's test, Greenhouse-Geisser correction was completed. For all statistical analyses of main and interaction effects, significance was set to α <0.05. Where significant main effects of Period were found, *post-hoc* comparisons with Bonferroni corrections were performed to determine pairwise differences (adjusting the α level for significance to 0.008, which is the result of dividing 0.05 by 6, which is the number of possible pairwise comparisons between the four periods).

## Results

**Figure 3:**
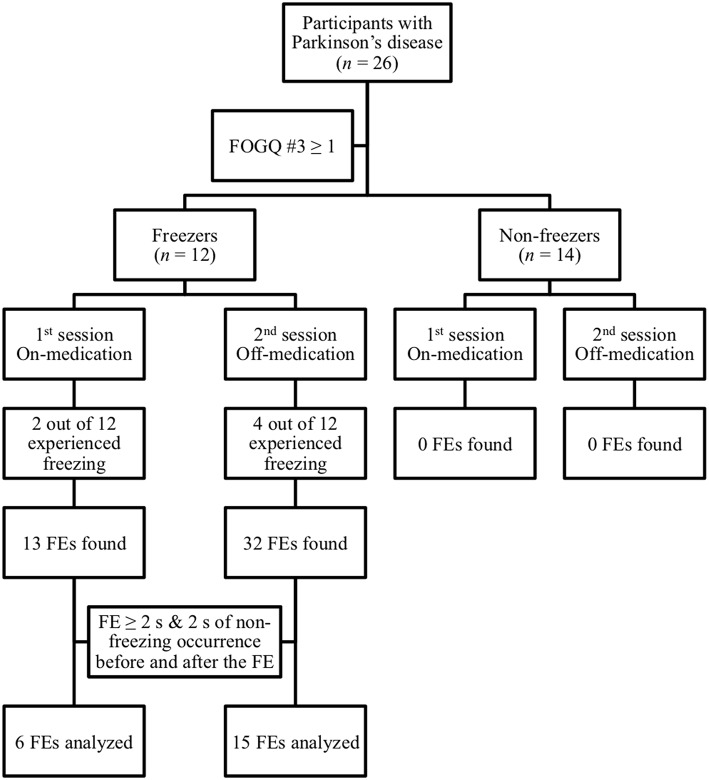
Schematic flow chart showing participants' categorization in freezers and non-freezers according to their self-reports scores of item 3 on the Freezing of Gait Questionnaire (FOGQ). Only the freezing episodes (FEs) lasting 2 s or more and with at least 2 s on non-freezing occurrence before and after (21 out of 45) were considered for analysis.

Participant characteristics and clinical evaluation scores are reported in Table 1. A total of 21 FEs (7.66 ± 4.90 s) were considered in the analysis of this study (see Figure 3). Those participants who experienced freezing in their on-state did also freeze off-medication, but the opposite did not occur.

**Table 1:** Participants' characteristics and clinical evaluation results (mean ± SD).

**Participants' characteristic**	**Freezers (*n* = 12)**	**Non-freezers (*n* = 14)**
Age (years)	69.1 ± 5.7	66.1 ± 7.0
Sex (m/w)	10/2	9/5
Height (cm)	171.8 ± 7.5	171.7 ± 8.4
MoCA (units)	27.7 ± 1.4	28.1 ± 1.6
UPDRS-III (on)	28.9 ± 9.8	21.7 ± 8.2
UPDRS-III (off)	37.9 ± 9.9	30.0 ± 10.7
H&Y (on)	2.7 ± 0.9	1.9 ± 0.7^*^
H&Y (off)	3.1 ± 1.0	2.3 ± 0.7
FOGQ (units)	10.3 ± 3.5	0.9 ± 1.4^*^
Disease Duration (years)	11.9 ± 5.3	3.9 ± 2.3^*^

*SD, standard deviation; m/w, men/women; cm, centimeters; MoCA, Montreal Cognitive Assessment; UPDRS-III, Unified Parkinson's Disease Rating Scale Part 3; H&Y, Hoehn and Yahr; FOGQ, Freezing of Gait Questionnaire*.

* *Significant difference between freezers and non-freezers*.

### Amplitude

Table 2 summarizes the EMG RMS amplitude values of the eight muscles across the four periods analyzed. There was a statistically significant effect of Period for most and least affected TA [respectively: $\chi_{\left(3\right)}^{2}$ = 16.03, *p* = 0.001, and $\chi_{\left(3\right)}^{2}$ = 11.80, *p* = 0.008], least affected GM [$\chi_{\left(3\right)}^{2}$ = 14.20, *p* = 0.003], and most and least affected BF [respectively: $\chi_{\left(3\right)}^{2}$ = 12.60, *p* = 0.006, and $\chi_{\left(3\right)}^{2}$ = 16.26, *p* = 0.001]. Pairwise comparisons show that on the least affected side, TA and GM activity around the FE (Before-FE, During-FE, and After-FE) was lower compared to Baseline (respectively: *Z* = −2.83, *p* = 0.005, *Z* = −3.32, *p* = 0.001). The opposite pattern was found at the least affected BF in which amplitude Before-FE, During-FE, and After-FE were significant higher compared to Baseline. Finally, in the most affected BF, amplitude was significantly lower Before-FE compared to Baseline (*Z* = −2.73, *p* = 0.006) but was similar to Baseline During-FE and After-FE.

**Table 2:** RMS amplitudes (Group means ± SD) during stepping-in-place task across four periods in individuals with Parkinson's disease who experienced freezing in the study.

**Muscle**	**Period main effect (*p* < 0.05)**	**Baseline (mV)**	**Before-FE (mV)**	**During-FE (mV)**	**After-FE (mV)**
Most affected RF	ns	0.071 ± 0.01	0.082 ± 0.03	0.079 ± 0.04	0.084 ± 0.03
Most affected BF	0.006	0.042 ± 0.02	0.060 ± 0.02^*^	0.057 ± 0.02	0.057 ± 0.02
Most affected TA	0.001	0.112 ± 0.05	0.093 ± 0.08	0.092 ± 0.09	0.090 ± 0.08
Most affected GM	ns	0.046 ± 0.02	0.049 ± 0.03	0.046 ± 0.03	0.048 ± 0.02
Least affected RF	ns	0.080 ± 0.04	0.081 ± 0.06	0.079 ± 0.05	0.086 ± 0.06
Least affected BF	0.001	0.036 ± 0.01	0.067 ± 0.03^*^	0.053 ± 0.02^#^	0.067 ± 0.03^&^
Least affected TA	0.008	0.144 ± 0.06	0.102 ± 0.10^*^	0.103 ± 0.10^#^	0.103 ± 0.10^&^
Least affected GM	0.003	0.059 ± 0.02	0.035 ± 0.02^*^	0.033 ± 0.02^#^	0.036 ± 0.02^&^

*RF, rectus femoris; BF, biceps femoris; TA, tibialis anterior; GM, gastrocnemius medialis; FE, freezing episode; mV, millivolts; ns, not significant*.

* *Significant difference between Baseline and Before-FE*.

# *Significant difference between Baseline and During-FE*.

& *Significant difference between Baseline and After-FE*.

### Variability

**Figure 4:**
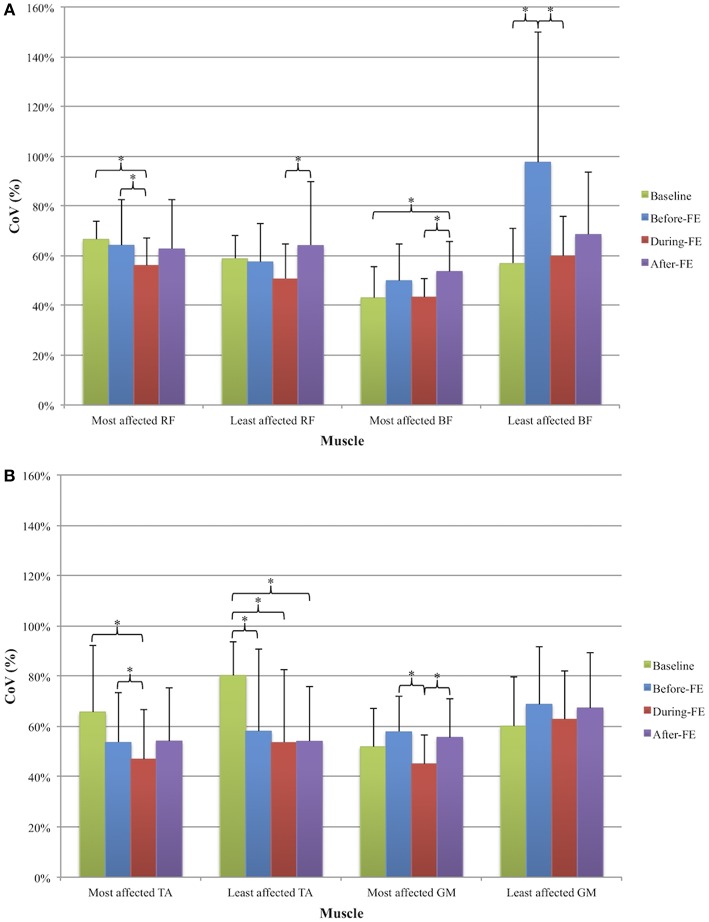
Average (+ SD) coefficient of variation (CoV, expressed as percentage) of the **(A)** proximal leg muscles [most and least affected rectus femoris (RF) and biceps femoris (BF)] and **(B)** distal leg muscles [most and least affected tibialis anterior (TA) and gastrocnemius medialis (GM)] across four periods: Baseline, 2s before a freezing episode (Before-FE), during the freezing episode (During-FE), and 2s after a freezing episode (After-FE). ^*^denotes significant pairwise differences (*p* < 0.008).

EMG variability significantly fluctuated across period in all muscles except in the least affected GM. There were significant Period effects in the most and least affected TA [respectively: $\chi_{\left(3\right)}^{2}$ = 11.29, *p* = 0.01, and $\chi_{\left(3\right)}^{2}$ = 21.97, *p* < 0.001], most affected GM [$\chi_{\left(3\right)}^{2}$ = 14.43, *p* = 0.002], most and least affected RF [respectively: $\chi_{\left(3\right)}^{2}$ = 8.49, *p* = 0.037, and $\chi_{\left(3\right)}^{2}$ = 9.69, *p* = 0.02], and most and least affected BF [respectively: $\chi_{\left(3\right)}^{2}$ = 11.11, *p* = 0.011, $\chi_{\left(3\right)}^{2}$ = 15.34, *p* = 0.002] (see Figure 4). Significant pairwise comparisons are reported in Figure 4. In all muscles, variability was the lowest During-FE. Baseline was the time at which variability of both TA was the highest, but at which variability of both BF was the lowest. In both TA, variability was lower at Before-FE compared to Baseline, and remained low until the end of recordings. Conversely, variability of both BF muscles was higher at Before-FE compared to Baseline, although it reached significance only for the least affected side. Finally, After-FE, variability was similar to Baseline in the most affected GM, the least affected BF, and in both RF.

### Functional Connectivity

Levels of MI within proximal and distal muscle pairs of both legs are reported in Table 3. A significant Period effect was found in the least affected TA and GM muscle pair [*F*_(3, 60)_ = 2.771, *p* = 0.049]. There was a trend for a decrease in MI from Baseline to Before-FE, with values staying constant afterwards, however, no significant differences were found in any pairwise comparisons across time.

**Table 3:** Mutual information (Group means ± SD) within lower limb muscle pairs during stepping-in-place task across four periods in individuals with Parkinson's disease who experienced freezing in the study.

**Muscle pair**	**Period main effect (*p* <0.05)**	**Baseline**	**Before-FE**	**During-FE**	**After-FE**
Most affected RF and BF	ns	0.198 ± 0.01	0.197 ± 0.02	0.193 ± 0.01	0.192 ± 0.01
Most affected TA and GM	ns	0.180 ± 0.02	0.173 ± 0.02	0.169 ± 0.02	0.171 ± 0.03
Least affected RF and BF	ns	0.187 ± 0.02	0.192 ± 0.01	0.192 ± 0.01	0.188 ± 0.02
Least affected TA and GM	0.049	0.168 ± 0.03	0.188 ± 0.02	0.190 ± 0.02	0.184 ± 0.02

*RF, rectus femoris; BF, biceps femoris; TA, tibialis anterior; GM, gastrocnemius medialis; FE, freezing episode; ns, not significant*.

## Discussion

The purpose of this study was to assess whether the occurrence of FEs could be explained by changes in EMG signal characteristics. Although a recent study reports EMG traces of distal muscles (TA and gastrocnemius lateralis) during and outside the occurrence of FOG (27), to our knowledge this is the first study to investigate a variety of muscle activity characteristics of the proximal muscles, as well as distal muscles, such as amplitude, variability, and functional connectivity, during the full spectrum of the freezing phenomenon, including immediately before and after the occurrence of the movement arrest. Our main findings are that a decrease in motor variability is seen during the occurrence of FEs. Moreover, we found that increased thigh muscles activity amplitude Before-FE compared to Baseline seem to compensate for FE-related changes in the activity patterns of shank muscles, which might contribute to the occurrence of freezing. However, EMG characteristics seen After-FE other than amplitude might contribute for overcoming freezing.

In our SIP task, EMG amplitude in the least affected TA muscle was significantly less than Baseline Before-FE, During-FE, and After-FE. These results are in line with those obtained by Nieuwboer et al. (25) who reported decreased amplitudes in TA EMG in the steps preceding an FE compared to normal walking or when preparing to stop. However, in their study, after normalizing the data for burst duration, increases in the EMG TA peaks were found. Authors interpret these increased TA amplitudes as a compensatory strategy of pulling the leg into swing, a gait phase that would most likely not necessitate TA contribution in the SIP task. Given that in their study, the burst durations became shorter in pre-fatigue gait cycles, these adaptations were likely insufficient in producing the desired toe clearance movements. In our study, BF muscles (especially the least affected) displayed the opposite change as compared to both TA across the four different periods (see Table 2). It is well-known that distal support muscles, such as TA, play an important role in postural stability during heel strike and loading response of the stance phase in a gait cycle (36). In fact, it has been shown that ankle dorsiflexors (e.g., TA) contribute to the backward angular momentum generated during the early stance phase of gait, preventing the body's center of mass from reaching an unstable position (37). Our results of EMG amplitude suggest that the decrease in the activation of both TA muscles before the FE may reduce stability, which is consistent with previous results showing that deficits in gait initiation in PD are a consequence of an abnormal activation timing and decrease activation of TA muscles (22–24) and that poor balance and falls in elderly are related to a loss of capabilities of strength and power of the ankle dorsiflexors (38, 39). Therefore, the amplitude decreases seen in TA muscles in our study could be compensated by increased activation of the BF on both sides. This compensatory increase in BF activation is more pronounced in the least affected side, suggesting a strategy of the freezers to rely on their more functional side. However, in this attempt of reaching postural stability, both BF muscles are functionally connected to both RF muscles, which maintain similar levels of activation during Baseline, Before-FE, During-FE, and After-FE. Therefore, our results suggest that while the RF is lifting the leg up during the SIP task, the BF is pulling the leg down, causing a simultaneous contraction of muscle pairs (RF and BF) that might explain the absence of movement seen when pwPD freeze.

Variations in spatial and temporal characteristics of gait have been previously investigated in order to better understand PD impairments. Increased spatiotemporal variability is considered one of the hallmarks of gait in PD (40–42) and even more so in freezers (28, 42–44). While the majority of studies of variability in pwPD quantified variability in gait events, kinematic, and kinetic patterns, much fewer studies have used EMG, which may help gain a better understanding of the muscular control mechanisms underlying motor variability. Nishikawa et al. (19) found that pwPD exhibited less variability in the right vastus lateralis amplitude activation than a healthy control group during a knee extension task. However, they also found a significantly higher variability of force exertion in the PD group. Moreover, Bailey et al. (14) suggested that a higher dysfunction during a gait task might be due to a higher variability of the gastrocnemius lateralis. In our study we found that variability of TA decreases toward the occurrence of freezing while it increases in the BF, with these changes being significant in the least affected side. Rather that defining an increase or decrease in muscle variability as beneficial or not, our results further support the idea of impaired muscle modulation in PD that has been previously described (12, 13). Moreover our results are in line with Siragy and Nantel (45) who proposed that the neuromuscular system is capable of processing certain amounts of variability. However, alterations below and above this range may interfere with gait stability and therefore lead to motor blockage or falls (43, 46–48). However, our results go a step further in suggesting that increased variability seen at some period of time in all muscles except both TA and the least affected GM, and seen even before the FE in both BF, may represent a strategy to compensate for the low TA variability that is maintained even after the FE. Thus, the ability to recover from a FE may rely in large part on the functioning of the proximal leg muscles and their ability to modulate their activity levels. Our study is the first to provide data to help explain how people may recover from a FE by accounting for the role of proximal muscles in modifying firing characteristics other than amplitude.

Lastly, the important role of proximal muscles in recovering from FEs is further reinforced by the increased functional connectivity between TA and GM before freezing, at a level that is maintained even after recovery from the episode. This suggests that distal muscles, when displaying signs of impaired function through the reduction of their respective amplitude and variability characteristics already before a FE, also increase their synergistic activity (functional connectivity), in a way that is not recovered even after the FE has ended. The fact that this is displayed more clearly in the distal muscles' least affected side is interesting and could further reinforce the use of muscles less impaired, and more able to help recover from a FE through their motor actions, such as proximal muscles, especially those on the least affected side. In fact, we found that in 81% of the cases, participants resumed the SIP with the most affected side, suggesting that they used the least affected side to stabilize their posture as a strategy to overcome freezing. However, this is speculation and would need to be explored further.

## Conclusion

In summary, our study reinforces the hypothesis of multi-muscle compensations for FEs that could be caused by impaired distal muscle function during gait in PD, and compensated by increases in the actions of proximal muscles (amplitude, variability), that help resume gait after a FE. PD is characterized by neuromuscular deficits, which might result in postural impairments and risk of falls, and our study is the first to show changes in electromyographical characteristics of proximal muscles, as well as distal leg muscles, before, during, and, after FEs. Since the number of FEs analyzed in this study is low, more work needs to be done with a larger sample of EMG data recorded during freezing in order to confirm our results and to further determine any role of medication on EMG patterns. Moreover, future studies looking into a larger number of muscles, not only from lower limb but also from the trunk, as well as other approaches to assess muscle co-contraction (such as cross-correlations) applied over movement cycles, are needed to provide further insight into the muscular mechanisms underlying freezing in PD.

## Data Availability

The datasets generated for this study are available on request to the corresponding author.

## Author Contributions

HC, JN, CP, and JC conceived and designed the study. HC carried out and executed the study. JN and JC supervised the work. HC and MM performed data analysis, data curation, and statistical analyses. HC wrote the first draft of the manuscript with revision and critiques by JN, CP, and JC. All authors read and approved the submitted version.

### Conflict of Interest Statement

The authors declare that the research was conducted in the absence of any commercial or financial relationships that could be construed as a potential conflict of interest.

## Abbreviations

pwPD: People with Parkinson's disease
FE(s): Freezing episode(s)
SIP: Stepping-in-place
RF: Rectus femoris
BF: Biceps femoris
TA: Tibialis anterior
GM: Gastrocnemius medialis.
